# Hepatocyte‐specific LRP1 silencing exacerbates brain amyloidosis without compensatory LDL receptor family involvement

**DOI:** 10.1002/alz70855_106122

**Published:** 2025-12-24

**Authors:** Brian Carson, Josephine Chu, Devaraj V. Chandrashekar, Jerome Garcia, Rachita K. Sumbria, Derick Han

**Affiliations:** ^1^ Keck Graduate Institute, Claremont, CA, USA; ^2^ School of Pharmacy, Chapman University, Irvine, CA, USA; ^3^ University of La Verne, La Verne, CA, USA

## Abstract

**Background:**

LDL receptor related protein 1 (LRP1) is a major hepatic receptor involved in lipoprotein metabolism, protease degradation, transmembrane receptor modulation, and clearance of excess protein, such as Aβ. Decreased expression of LRP1 due to liver injury can impair receptor‐mediated clearance, increasing Aβ availability in the periphery. We investigated the effects of hepato‐specific silencing of LRP1 on Aβ deposition in the brain. Additionally, other LDL family members (LRP5, LRP6, and LDLR) that may participate in Aβ clearance were monitored in the liver to ensure non‐compensatory effects of LRP1 silencing.

**Method:**

4‐month‐old male double transgenic (APP/PS1) AD mice were injected with adeno‐associated virus 8 (AAV8) containing microRNA targeting LRP1 (LRP1‐silenced) or LacZ (control). (*n* = 6‐7 per group). Organs were harvested 12‐ and 28‐weeks post injection, homogenized and assayed using western blotting. Western blots of liver homogenates were probed for LDL receptor related proteins (LDLR, LRP1, LRP5, LRP6), receptors involved in Aβ clearance (RAGE, MDR1), and AD biomarkers (APOE). Beta‐actin and GDH were used as loading controls. Brain and liver Aβ were quantified using sandwich enzyme‐linked immunosorbent assays (ELISA).

**Result:**

Hepatic LRP1 was silenced >80% in both 12‐ and 28‐week mouse samples (*p*≤0.05) compared to AAV8 LacZ control. Hepato‐specific LRP1 silencing significantly increased TBS‐soluble mouse (mAβ42) and human (hAβ42) amyloid beta levels in the brain and hAβ42 in the periphery (*p*≤0.05). No significant change in hepatic MDR1 or APOE expression was observed. Hepatic APP showed a non‐significant increasing trend in expression with LRP1 silencing. Hepatic LDL receptor family proteins did not show significant changes at 12‐ or 28‐weeks.

**Conclusion:**

Hepatic LRP1‐mediated clearance of peripheral Aβ plays an important role in mitigating brain amyloidosis. Hepatic downregulation of LRP1 in AD mice correlates with increased brain amyloidosis that is neither the result of compensation in other hepatic LDL receptors nor changes in receptors involved in Aβ clearance. Hepatic LRP1 specifically may hold promise as a therapeutic target, especially in conditions resulting in liver damage and hepatic LRP1 reduction such as obesity and alcoholism, known modifiable risk factors for AD.

